# High-fat diet activates liver iPLA_2_γ generating eicosanoids that mediate metabolic stress

**DOI:** 10.1016/j.jlr.2021.100052

**Published:** 2021-02-24

**Authors:** Sung Ho Moon, Beverly Gibson Dilthey, Xinping Liu, Shaoping Guan, Harold F. Sims, Richard W. Gross

**Affiliations:** 1 Division of Bioorganic Chemistry and Molecular Pharmacology, Department of Medicine, Washington University School of Medicine, Saint Louis, MO, USA; 2 Department of Developmental Biology, Washington University School of Medicine, Saint Louis, MO, USA; 3 Center for Cardiovascular Research, Department of Medicine, Washington University School of Medicine, Saint Louis, MO, USA; 4 Department of Chemistry, Washington University, Saint Louis, MO, USA

**Keywords:** eicosanoids, phospholipases A_2_, diet and dietary lipids, obesity, mitochondria, mitochondrial respiration, mitochondrial permeability transition pore, hydroxyeicosatetraenoic acids, hepatocyte, cell death, AA, arachidonic acid, AMPP, N-(4-aminomethylphenyl) pyridinium, ANT, adenine nucleotide translocase, EET, epoxyeicosatrienoic acid, FCCP, 2-[2-[4-(trifluoromethoxy)phenyl]hydrazinylidene]-propanedinitrile, FFA, free fatty acid, HEPiPLA_2_γKO, hepatocyte-specific iPLA_2_γ-knockout, HETE, hydroxyeicosatetraenoic acid, HF, high-fat, iPLA_2_γ, calcium-independent phospholipase A_2_γ, LDH, lactate dehydrogenase, LOX, lipoxygenase, mPTP, mitochondrial permeability transition pore, NC, normal-chow, TAG, triacylglycerol, NDGA, nordihydroguaiaretic acid

## Abstract

High-fat (HF) diet–induced obesity precipitates multiple metabolic disorders including insulin resistance, glucose intolerance, oxidative stress, and inflammation, resulting in the initiation of cell death programs. Previously, we demonstrated murine germline knockout of calcium-independent phospholipase A_2_γ (iPLA_2_γ) prevented HF diet–induced weight gain, attenuated insulin resistance, and decreased mitochondrial permeability transition pore (mPTP) opening leading to alterations in bioenergetics. To gain insight into the specific roles of hepatic iPLA_2_γ in mitochondrial function and cell death under metabolic stress, we generated a hepatocyte-specific iPLA_2_γ-knockout (HEPiPLA_2_γKO). Using this model, we compared the effects of an HF diet on wild-type versus HEPiPLA_2_γKO mice in eicosanoid production and mitochondrial bioenergetics. HEPiPLA_2_γKO mice exhibited higher glucose clearance rates than WT controls. Importantly, HF-diet induced the accumulation of 12-hydroxyeicosatetraenoic acid (12-HETE) in WT liver which was decreased in HEPiPLA_2_γKO. Furthermore, HF-feeding markedly increased Ca^2+^ sensitivity and resistance to ADP-mediated inhibition of mPTP opening in WT mice. In contrast, ablation of iPLA_2_γ prevented the HF-induced hypersensitivity of mPTP opening to calcium and maintained ADP-mediated resistance to mPTP opening. Respirometry revealed that ADP-stimulated mitochondrial respiration was significantly reduced by exogenous 12-HETE. Finally, HEPiPLA_2_γKO hepatocytes were resistant to calcium ionophore-induced lipoxygenase-mediated lactate dehydrogenase release. Collectively, these results demonstrate that an HF diet increases iPLA_2_γ-mediated hepatic 12-HETE production leading to mitochondrial dysfunction and hepatic cell death.

Obesity is a major health threat to modern societies through facilitating multiple pathologic processes such as hypertension, congestive heart failure, and type 2 diabetes mellitus (1). Murine models of dietary-induced obesity have been extensively studied to understand the pathophysiological effects of human obesity. However, responses to different high-fat (HF) diets are often variable and mouse-strain dependent (2, 3, 4, 5). Saturated fatty acid-enriched diet–induced obesity commonly results in nonalcoholic fatty liver disease, hyperinsulinemia, impaired glucose tolerance, hypertension, and hepatic cell death through chronic disorders of lipid metabolism. Further, oxidative stress in the early stages of the obese state is known to contribute to the accumulation of toxic oxidized lipids, uncontrolled opening of the mitochondrial permeability transition pore (mPTP), the synthesis of proinflammatory cytokines, and initiation of cell death programs (6, 7, 8).

Growing evidence suggests that biologically active oxidized arachidonic acid (AA) metabolites (i.e., eicosanoids) are generated upon cellular oxidative stress causing the metabolic dysfunction in disease states such as nonalcoholic fatty liver disease, cardiovascular disease, and diabetes (9, 10, 11). For example, Zhang *et al.* (10) have demonstrated that 12-hydroxyeicosatetraenoic acid (12-HETE), which is produced by 12-lipoxygenase (12-LOX), was generated during hepatic ischemia/reperfusion injury resulting in inflammation that could be attenuated by inhibition of 12-LOX enzymatic activity. Other studies have reported the significant accumulation of eicosanoids during cardiac ischemia/reperfusion leading to progression of heart failure (12, 13, 14, 15). In addition, hydroxyeicosatetraenoic acids (HETEs) have been identified by a number of studies as proinflammatory signaling molecules to regulate the innate immune response by activation of mature cytokine release through G-protein–coupled receptors (9, 16, 17).

Since production of eicosanoids is typically initiated by phospholipases A_2_ which release AA from phospholipids for multiple oxygenases such as lipoxygenases (LOXs), cyclooxygenases (COXs), and cytochrome P450 epoxygenases, modulation of cellular lipid oxidation by cellular phospholipases has been a topic of substantial interest. Previously, we reported that loss of calcium-independent phospholipase A_2_γ (iPLA_2_γ) activity in the germline iPLA_2_γ knockout mouse resulted in its resistance to HF-induced weight gain and maintenance of insulin sensitivity. However, the precise mechanisms and mediators responsible for the observed mitochondrial dysfunction were not identified. Additionally, we demonstrated that global genetic ablation of iPLA_2_γ prevented pathologic Ca^2+^-induced opening of the mPTP concomitant with a decrease in apoptotic cytochrome *c* release into the extramitochondrial space (18). Furthermore, we identified the accumulation of oxidized AA metabolites after cardiac ischemia/reperfusion injury (15). Importantly, these metabolites were able to accelerate Ca^2+^-mediated dissipation of mitochondrial membrane potential as determined using isolated myocardial mitochondria. Remarkably, cardiac myocyte–specific knockout of iPLA_2_γ reduced infarct size with attenuation of toxic eicosanoid production following ischemia-reperfusion (15). Collectively, these studies suggested that pathologic iPLA_2_γ activation and/or overexpression in a pathologic state facilitates production of deleterious eicosanoids promoting apoptotic/necrotic cell death and human heart failure.

In this study, we engineered tissue-specific gene knockouts using the Cre/Lox system to generate a hepatocyte-specific iPLA_2_γ knockout (HEPiPLA_2_γKO) mouse which enabled us to investigate the specific roles of iPLA_2_γ in liver and its impact on hepatic mitochondrial function. Herein, we demonstrate that hepatic KO of iPLA_2_γ dramatically reduced 12-HETE production induced by HF feeding resulting in the desensitization of mPTP opening to Ca^2+^ activation and preservation of ADP-dependent inhibition of mPTP opening. This study collectively demonstrates that hepatic iPLA_2_γ is a central regulator of diet-dependent production of lipid mediators in hepatocytes and mitochondrial dysfunction during pathologic disease states contributing to hepatocyte cell death pathways.

## Materials and methods

### Materials

Rotenone, ADP, antimycin A, L-glutamic acid, L-malic acid, succinate disodium, fatty acid–free BSA, collagenase type IV, Percoll®, and glucose were purchase from Millipore Sigma Corp. Calcium ionophore A23187, N-(methylsulfonyl)-2-(2-propynyloxy)-benzenehexanamide, nordihydroguaiaretic acid (NDGA), ibuprofen, oligomycin, cell culture supplements, thromboxane B_2_-d_4_, prostaglandin E_2_-d_4_, 12-HETE-d_8_, and 2-[2-[4-(trifluoromethoxy)phenyl]hydrazinylidene]-propanedinitrile (FCCP) were purchased from Cayman Chemical Company. The lactate dehydrogenase (LDH)-cytotoxicity assay kit, HepatoZYME-SFM, and other chemicals for preparation of buffers for mitochondrial isolation, respiration, and swelling were obtained from ThermoFisher Scientific Inc. A rabbit polyclonal anti-iPLA_2_γ antibody was generated in our laboratory as described previously (15).

### Animal diets and study protocols

Mice were maintained and used in strict accordance with the National Institutes of Health guidelines for humane treatment of animals. All protocols were reviewed and approved by the Institutional Animal Care and Use Committee of Washington University. Mice were fed a normal-chow (NC) diet (LabDiet, Cat. #5053). For HF feeding, mice at the age of 3 months were fed a Western Diet with 42% kcal from fat (Envigo, Cat. #TD.88137) for 12–14 weeks.

### Generation of the hepatocyte-specific iPLA_2_*γ* knockout mouse

**Fig. 1:**
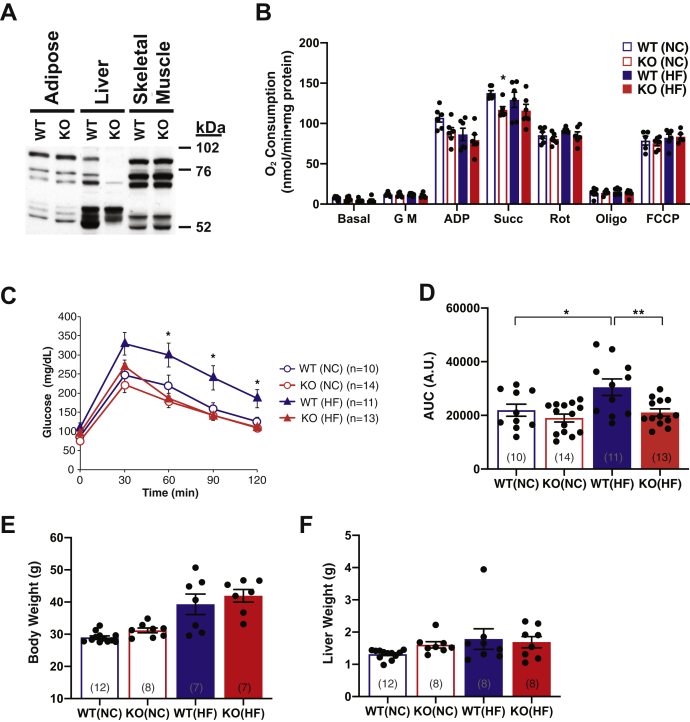
Tissue-specific iPLA_2_γ expression analysis, mitochondrial respiration rates, and glucose tolerance test results of wild-type (WT) versus hepatocyte-specific iPLA_2_γ KO (KO) mice fed a normal-chow or high-fat diet. A: Hepatocyte-specific iPLA_2_γKO mice were generated as described in Materials and Methods, and iPLA_2_γ protein expression levels in mitochondria from multiple organs were determined by Western blot analysis. Isoforms of iPLA_2_γ (i.e., 85, 74, 63, 52 kDa bands) were ablated specifically in liver in comparison to adipose and skeletal muscle tissues. B: High resolution respirometry of hepatic mitochondria isolated from six WT and six HEPiPLA_2_γKO mice (∼6–7 month-old) after normal-chow (NC) or high-fat (HF) feeding for 12 weeks. Mitochondrial respiration states were observed by the sequential addition of substrates and inhibitors as indicated in the figure: Basal (mitochondria alone), G M (glutamate/malate), ADP, Succ (succinate), Rot (rotenone), Oligo (oligomycin), and FCCP. Antimycin A was finally added to determine oxygen consumption by nonoxidative phosphorylation reactions which was then subtracted from each prior condition measured. ∗*P* < 0.05 when compared with WT on the same diet. C: Glucose tolerance test (GTT) results from WT and HEPiPLA_2_γKO mice after 3 months of high-fat (HF) feeding which was started at 3 months of age. Mice were then challenged with a bolus intraperitoneal injection of glucose at t = 0, and blood glucose levels were monitored at 30 min intervals for 2 h. The results indicate that hepatocyte-specific knockout of iPLA_2_γ significantly improves glucose tolerance. Area under the curve (AUC) of GTT curve is presented in D. Values are expressed as means with SEM. ∗*P* < 0.05 and ∗∗*P* < 0.01. Body weights (E) and wet liver weights (F) of WT and KO mice after normal-chow or high-fat feeding for 12 weeks were measured for comparison. Animal numbers used for each of these measurements are indicated in the brackets for each graph bar in the figure. HEPiPLA_2_γKO, hepatocyte-specific iPLA_2_γ-knockout; iPLA_2_γ, calcium-independent phospholipase A_2_γ.

To definitively identify the mechanistic importance of iPLA_2_γ in hepatocytes, we engineered a mouse strain bearing a liver-specific knockout of iPLA_2_γ. Because of the presence of multiple transcriptional start sites in iPLA_2_γ, our strategy was to flox exon 5 (encoding the iPLA_2_γ active site) and ablate it by crossing with a transgenic mouse expressing Cre recombinase under the control of a minimal mouse albumin promoter (MMAP) (The Jackson Laboratory, Cat. # 003574). Hepatocyte-specific knockout of iPLA_2_γ was confirmed by Western blot analyses to determine iPLA_2_γ protein expression levels in mitochondria isolated from WT and HEPiPLA_2_γKO liver, white adipose, and skeletal muscle tissues. Western blot analysis in comparisons with MMAP-Cre WT/WT nonfloxed mouse tissues demonstrated the specific ablation of iPLA_2_γ in liver mitochondria but not in mitochondria from other tissues in the HEPiPLA_2_γKO mouse (Fig. 1).

### Isolation of Hepatic Mitochondria

Liver mitochondria were isolated by differential centrifugation as previously described (18). Briefly, the excised liver tissue from WT and HEPiPLA_2_γKO (∼6–7 month old) male mice euthanized by cervical dislocation was immediately washed in ice-cold mitochondrial isolation buffer (MIB: 0.21 M mannitol, 70 mM sucrose, 0.1 mM potassium-EDTA, 1 mM EGTA, 10 mM Tris-HCl, 0.5% BSA, pH 7.4) and cut into small pieces with a razor blade. Pieces of liver tissue were homogenized via 10 strokes of a Teflon homogenizer using a rotation speed of 120 rpm at 4°C ambient temperature. The homogenate was centrifuged for 7 min at 850 *g*, and the resultant supernatant was centrifuged at 12,000 *g* for 10 min. The pellet was resuspended in mitochondrial isolation buffer without BSA and centrifuged at 7,500 *g* for 10 min. The pellet was resuspended in MIB without BSA and stored on ice until use for various experiments. Mitochondrial protein content was determined using a BCA protein assay using BSA as a standard.

### Mitochondrial High Resolution Respirometry

Mitochondrial high resolution respirometry was performed utilizing an OROBOROS® Oxygraph 2K respirometer (Innsbruck, Austria). Isolated liver mitochondria (100 μg) were resuspended into mitochondrial respiration buffer (MiR05: 110 mM sucrose, 60 mM potassium lactobionate, 20 mM taurine, 20 mM Hepes, 10 mM KH_2_PO_4_, 3 mM MgCl_2_, 0.5 mM EGTA, 1% BSA (Fraction V), pH 7.1) in the 2-ml chambers of the respirometer. The oxygen concentration and O_2_ consumption rate at each respiratory state were monitored with sequential addition of substrates and inhibitors in the order as indicated in the figures: 10 mM glutamate/5 mM malate (state 2), 1.25 mM ADP (state 3), 5 mM succinate (state 3_max_), 1 μM rotenone, 1 μM oligomycin (state 4o, oligomycin-induced state 4), and 1 μM FCCP (state 3u, uncoupling). For the experiments with 12-HETE, mitochondria were placed into MiR05 buffer containing 5 μM 12-HETE in the respirometer chambers followed by sequential addition of substrates and inhibitors. Oxygen consumption was calculated as a time derivative of oxygen concentration using DatLab 6.0 Analysis software (OROBOROS®, Innsbruck, Austria).

### Mass spectrometric analysis of lipids

Hepatic tissue from WT and HEPiPLA_2_γKO mice was excised after euthanasia by cervical dislocation and immediately flash-frozen in liquid nitrogen. Lipids from hepatic tissue and primary hepatocytes were extracted using a modified Bligh and Dyer procedure. For the determination and quantitation of eicosanoids, the extracted eicosanoids were further enriched by solid phase extraction, derivatized using a charge-switch strategy with N-(4-aminomethylphenyl) pyridinium (AMPP), and analyzed by LC-MS/MS with multiple-reaction monitoring using an LTQ-Orbitrap mass spectrometer (Thermo Scientific, San Jose, CA) as described previously (19). Mass spectrometric analyses of phospholipids, triacylglycerols (TAGs), and free fatty acid (FFA) present in isolated hepatic tissues were performed utilizing a TSQ Quantum Ultra triple-quadrupole mass spectrometer (Thermo Scientific, San Jose, CA) equipped with an automated nanospray apparatus, Triversa NanoMate (Advion, Inc., Ithaca, NY) as previously described (20, 21). For the quantitation of FFAs, fatty acids extracted from hepatic tissue using a modified Bligh and Dyer procedure were derivatized with AMPP and analyzed by a TSQ mass spectrometer in the positive-ion mode as previously described (21).

### Isolation and culture of primary mouse hepatocytes

Murine hepatocytes were isolated by enzymatic digestion via liver perfusion as described previously with minor modifications (22). All media used for liver perfusion were kept at 40°C using a water bath and delivered by peristaltic pump. Mice were anesthetized by intraperitoneal injection of a ketamine (100 mg/kg) and xylazine (10 mg/kg) mixture and placed in a supine position on a tray. The cannula was inserted into the portal vein of the liver site which was flushed with warm perfusion medium I {500 ml PBS [pH 7.4], 5 ml of sterile Buffer A [1 M Hepes (pH 7.4) and 5% KCl (w/v)], 2.5 ml of 1 M glucose, 0.5 ml 200 mM EDTA} at 6 ml/min followed by cutting of the inferior portal vein to allow drainage of the perfusate while clamping the aorta from the heart. After 8 min of perfusion with perfusion medium I, perfusion media II [500 ml PBS (pH 7.4), 5 ml of sterile Buffer A, 10 ml of 1 M Hepes, 2.5 ml of 1 M sterile glucose solution, 1 ml of 500 mM CaCl_2_] containing collagenase type IV (0.07–0.08 mg/ml) was delivered at 6 ml/min for 4 min. The liver was then excised after collagenase digestion and placed in cold perfusion medium II. The lobes of the liver were gently torn apart to release free hepatocytes into the media. Hepatocytes were collected through a cell strainer (40 μm) and washed twice with attachment medium [Williams' E medium containing 1% (v/v) penicillin/streptomycin, 1% (v/v) 200 mM L-glutamine, 1% (v/v) nonessential amino acid, and 10% heat-inactivated fetal bovine serum] by centrifugation at 50 *g* at 4°C. The cells were then resuspended in the media containing attachment medium and 85% Percoll™ in PBS (1:1, v/v) and centrifuged at 200 *g* for 10 min. The supernatant with floating dead cells was discarded, and the cell pellet was washed 3 times with the attachment medium. Cell viability was monitored by trypan blue exclusion which routinely indicated >85% viability. The hepatocytes in the attachment medium were plated on cell culture dishes or well plates and incubated for 3 h before exchanging this media with cell culture medium (serum-free HepatoZYME-SFM containing 1% penicillin/streptomycin and 200 mM L-glutamine). Primary hepatocytes were utilized for experiments within 2 days after isolation.

### Determination of mitochondrial swelling

Mitochondrial swelling assays were performed to determine mPTP opening as previously described (18). Briefly, isolated intact hepatic mitochondria from WT and HEPiPLA_2_γKO mice were placed in mitochondrial swelling buffer [3 mM Hepes buffer (pH 7.0) containing 0.23 M mannitol, 70 mM sucrose, 5 mM succinate, 1 μM rotenone and 1 mM KH_2_PO_4_] and equilibrated for 10 min at 23°C. Mitochondrial swelling was initiated by adding Ca^2+^ at the indicated concentrations at 25°C. For ADP inhibition experiments, ADP at the indicated concentrations was added to the mitochondrial swelling buffer containing mitochondria before initiating swelling with Ca^2+^. For experiments examining the effect of various HETEs, mitochondria were preincubated with purified commercially prepared HETEs at the indicated concentrations [delivered in DMSO vehicle (1%, v/v, final concentration)] before initiating mitochondrial swelling by addition of Ca^2+^. EGTA (10 μM) was used as a negative control (without Ca^2+^) for Ca^2+^-induced mitochondrial swelling. Inhibition of mitochondrial swelling with cyclosporin A (2 μM) was used to determine mitochondrial swelling specific for mPTP opening. Decreases in the absorbance (540 nm) of the mitochondria were measured every 15 s using a SpectraMax M5e microplate reader (Molecular Devices, Sunnyvale, CA).

### Determination of hepatocyte cytotoxicity

Hepatocyte cellular cytotoxicity was measured by determining the catalytic activity of released LDH using a Cytotoxicity Assay Kit (ThermoFisher Scientific). Briefly, hepatocytes isolated from WT and HEPiPLA_2_γKO mouse liver as described above were allowed to attach to 12-well plates (0.15 × 10^6^ cells/well) for 2 days and then incubated in serum-free and phenol red-free Williams' E medium for 3 h. The attached cells were then exposed to DMSO vehicle alone (0.1%, v/v) or 5 μM A23187 for the indicated times. After stimulation with calcium ionophore, the cell media was collected, briefly centrifuged, and the resultant supernatant was collected for assay of LDH activity as described by the manufacturer's instructions. Maximum LDH release from the hepatocytes was determined by incubation of the attached cells with media containing 0.5% Triton X-100 for 1 min followed by measurement of LDH activity in the media.

### Glucose tolerance test

Wild-type and HEPiPLA_2_γKO mice (6–7 months old) fed a NC or an HF diet for 12 weeks were fasted overnight (16–18 h) on wood chip bedding with ad libitum access to water. Blood was drawn from the tail vein the following day before intraperitoneal injection of glucose (2 mg/g body weight). Blood from tail vein was then collected at 30, 60, 90, and 120 min after intraperitoneal glucose injection. Blood glucose levels were immediately measured using a glucose meter with test strips. The area under the curve was calculated by using Prism version 8.4.1 purchased from GraphPad Software, LLC.

### Statistical analyses

A unpaired Student *t* test was performed to determine the significance of differences between two groups. A two-tailed *P*-value less than 0.05 was considered significant. All data were presented as means ± SEM.

## Results

### Hepatocyte-specific iPLA_2_γ-knockout mice exhibited higher glucose clearance rates after HF feeding than WT controls

To gain insight into the specific roles of hepatic iPLA_2_γ in the integration of organismal energy metabolism, glucose utilization, and mitochondrial bioenergetics after HF feeding, we engineered and generated a HEPiPLA_2_γKO. To confirm the tissue-specific deletion of hepatocyte iPLA_2_γ, Western blot analyses to determine the expression levels of different isoforms of iPLA_2_γ in multiple tissues including adipose, skeletal muscle, and liver were performed. Isoforms of iPLA_2_γ at 85, 74, 63, and 52 kDa were nearly completely absent in hepatic mitochondria of the HEPiPLA_2_γKO mouse. In contrast, there were no significant changes in the expression levels of these iPLA_2_γ isoforms in adipose and skeletal muscle tissues in comparison to WT, thereby identifying the specificity of genetic knockout of iPLA_2_γ in liver (Fig. 1A). Because iPLA_2_γ is known to be localized to mitochondrial membranes, we next determined if mitochondrial respiration was altered in isolated HEPiPLA_2_γKO liver mitochondria. Mitochondrial oxygen consumption utilizing glutamate/malate as substrate was modestly decreased in HEPiPLA_2_γKO liver mitochondria in comparison to their WT counterparts on a NC diet. In addition, liver mitochondria isolated from HF-fed WT mice demonstrated a modest decrease in state 3 respiration relative to NC-fed WT controls, although overall mitochondrial respiratory rates were not significantly altered by HF feeding (Fig. 1B). Hepatic mitochondrial oxygen consumption rates of HEPiPLA_2_γKO mice were not different from those of WT littermates after HF feeding. Similar to the global iPLA_2_γ KO mouse (20), HEPiPLA_2_γKO mice notably exhibited improved glucose tolerance relative to WT controls following HF feeding (Fig. 1C, D). In contrast to the global iPLA_2_γ KO mouse (which is resistant to an HF diet-induced weight gain) (20), the body and wet liver weights of HF-fed HEPiPLA_2_γKO mice were not significantly different from those of WT controls on an HF diet (Fig. 1E, F).

### HF feeding induced the accumulation of fatty acids including AA, a precursor of eicosanoids, in WT liver which was markedly attenuated in HEPiPLA_2_*γ*KO mice

**Fig. 2:**
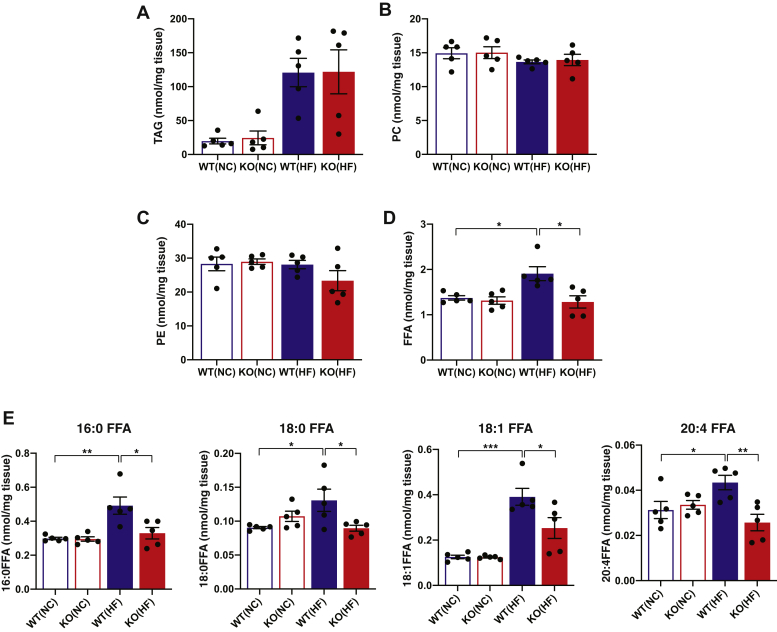
Contents of total triacylglycerols, select phospholipids, and nonesterified fatty acids present in wild-type and hepatocyte-specific iPLA_2_γ KO liver tissue after normal-chow or high-fat feeding. Liver tissue was excised from five wild-type (WT) and five HEPiPLA_2_γKO (KO) mice (∼6–7 months of age after normal-chow or high-fat feeding for 12 weeks), and lipids were extracted by a modified Bligh-Dyer procedure. Mass spectrometric analyses were performed as described in Materials and Methods to determine the quantities of total triacylglycerol (TAG) (A), phosphatidylcholine (PC) (B), phosphatidylethanolamine (PE) (C) and free fatty acid (FFA) (D) molecular species. The quantities of predominant liver fatty acid molecular species are shown in E. Mean values are presented with SEM. ∗*P* < 0.05, ∗∗*P* < 0.01, and ∗∗∗*P* < 0.001. HEPiPLA_2_γKO, hepatocyte-specific iPLA_2_γ-knockout; iPLA_2_γ, calcium-independent phospholipase A_2_γ.

Western diets rich in saturated fatty acids induce a large accumulation of FFAs and TAGs containing saturated fatty acyl groups, which eventually lead to increased oxidative stress and dietary-induced obesity. Mass spectrometric lipid analyses showed that an HF diet led to the dramatic accumulation of TAGs in both WT and HEPiPLA_2_γKO mouse livers. Furthermore, the amounts of TAGs in HEPiPLA_2_γKO mouse liver were not significantly different from those present in WT controls (Fig. 2A). The major lipid components of membrane bilayers (i.e., phospholipids including phosphatidylcholine and phosphatidylethanolamine molecular species) were not significantly altered between either WT versus HEPiPLA_2_γKO fed either a normal diet or an HF diet (Fig. 2B, C). Importantly, the content of hepatic FFAs in WT mice fed an HF diet was higher than that present in NC-fed mice. In stark contrast, genetic deletion of hepatic iPLA_2_γ in the HEPiPLA_2_γKO mouse effectively abolished HF diet–induced increases in nonesterified fatty acids (Fig. 2D, E). Similarly, the HF diet–induced elevation of AA, a precursor of various signaling lipid metabolites, in WT mice was completely absent in HEPiPLA_2_γKO mouse liver.

### High-fat feeding induced the accumulation of 12-HETE in WT liver which was markedly attenuated in HEPiPLA_2_*γ*KO mice

**Fig. 3:**
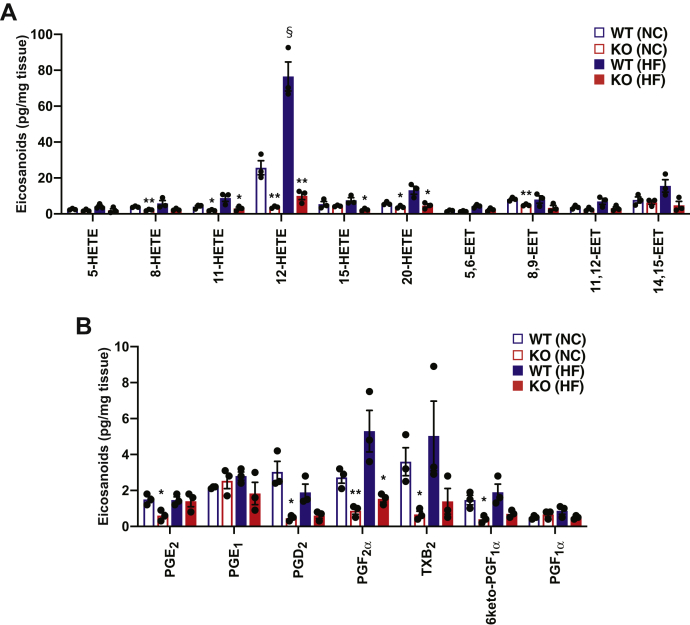
Eicosanoid profiles in wild-type and hepatocyte-specific iPLA_2_γKO liver tissue after normal-chow or high-fat feeding. Eicosanoids in wild-type (WT) and HEPiPLA_2_γKO (KO) mouse livers after normal-chow or high-fat feeding were extracted and derivatized with AMPP. Mass spectrometric analyses for the identification and quantification of eicosanoids including HETEs, EETs (A), and prostaglandins (B) were performed by LC-MS/MS via MRM in the positive-ion mode with accurate mass analysis of diagnostic product ions following separation of molecular species using a reverse phase column as described under Materials and Methods. ∗*P* < 0.05 and ∗∗*P* < 0.01 when compared with WT on the same diet. §*P* < 0.005 in comparisons of WT NC versus HF. EET, epoxyeicosatrienoic acid; HEPiPLA_2_γKO, hepatocyte-specific iPLA_2_γ-knockout; HETE, hydroxyeicosatetraenoic acid; HF, high-fat; MRM, multiple-reaction monitoring; NC, normal-chow; PG, prostaglandin; TXB_2_, thromboxane B_2_.

Oxidized AA metabolites are well known to be generated during oxidative stress acting as a pathologic insult to multiple organ systems. To assess whether hepatic oxidized AA metabolic profiles are influenced by an HF diet, we determined the levels of multiple eicosanoids in WT and HEPiPLA_2_γKO mouse liver after NC or HF feeding for 12 weeks. Eicosanoids extracted from liver were enriched by solid phase extraction, derivatized with AMPP and their quantities were determined by high-resolution accurate-mass mass spectrometry as described in “Materials and Methods.” During HF feeding, various eicosanoids including HETEs, epoxyeicosatrienoic acids (EETs), and prostaglandins were found to increase to varying degrees (Fig. 3). Among the identified eicosanoids, 12-HETE was the most abundant eicosanoid molecular species in WT mice regardless of the type of diet. Importantly, HF feeding induced the accumulation of 12-HETE in WT liver by ∼2.5 fold when compared with the NC-fed WT mouse. However, 12-HETE levels in NC-fed HEPiPLA_2_γKO mouse liver were only modestly higher than other HETE molecular species (Fig. 3). Furthermore, HF feeding did not cause accumulation of hepatic12-HETE in the HEPiPLA_2_γKO mouse in comparison to WT. These results indicate that hepatic iPLA_2_γ plays a predominant role in determining HF diet–induced increases in 12-HETE and other eicosanoids (e.g., 20-HETE, 14,15-EET, PGF_2_α, and thromboxane B_2_).

### HETEs enhance Ca^2+^-induced mPTP opening in liver mitochondria

**Fig. 4:**
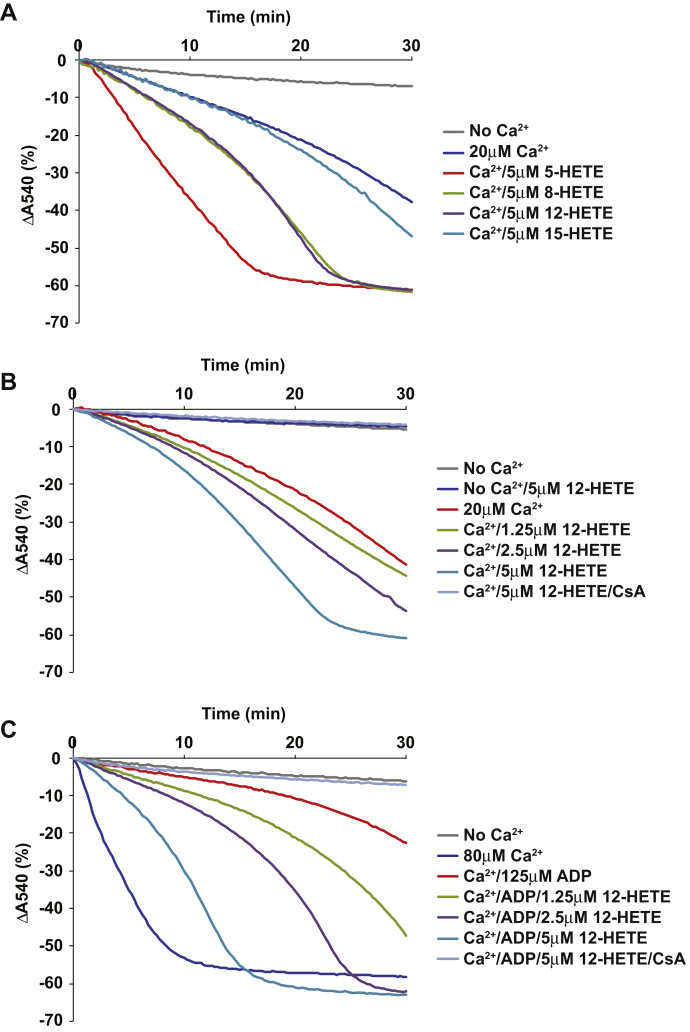
HETE-facilitated Ca^2+^-induced opening of the hepatic mitochondrial permeability transition pore. Measurements of mitochondrial swelling were performed utilizing mitochondria isolated from wild-type mouse liver. A: Mitochondrial swelling initiated by 20 μM Ca^2+^ in the absence or presence of various HETEs (including 5-, 8-, 12-, 15-HETE) at 5 μM final concentration. B: 12-HETE-mediated facilitation of mPTP opening was determined by performing Ca^2+^-induced (20 μM) mitochondrial swelling in the presence of 0 (DMSO vehicle alone), 1.25, 2.5, or 5 μM 12-HETE. 12-HETE-facilitated mitochondrial swelling was inhibitable with 2 μM cyclosporin A (CsA) indicative of a conventional cyclophilin D-mediated mPTP opening process. C: ADP (125 μM)–mediated inhibition of mitochondrial swelling activated by 80 μM Ca^2+^ as measured in the absence (DMSO vehicle alone) or presence of increasing concentrations of 12-HETE (1.25, 2.5, or 5 μM). 12-HETE reversed ADP-inhibited mitochondrial swelling in a cyclosporine A-sensitive way indicating cyclophilin D-dependent mPTP opening. Representative tracings from six independent preparations are shown in the figure. HETE, hydroxyeicosatetraenoic acid; mPTP, mitochondrial permeability transition pore.

Previously, we reported that hepatic mitochondria from the germline iPLA_2_γ KO mouse were resistant to Ca^2+^-induced mPTP opening (18). Because 12-HETE was the most abundant eicosanoid increased by HF feeding, the effect of 12-HETE on mPTP opening was investigated using liver mitochondria isolated from NC-fed mice. First, mitochondria were preincubated in the absence or presence of various HETEs (5 μM final concentration), including 5-, 8-, 12-, or 15-HETE. Next, mitochondrial swelling was induced by addition of 20 μM Ca^2+^ (final concentration). The results indicated that most of the HETEs tested (5-HETE, 8-HETE, 12-HETE) markedly accelerated mitochondrial swelling with the rank order of potency to activate mPTP opening at 5 μM as: 5-HETE > 12-HETE ≅ 8-HETE (Fig. 4A). 5-HETE was the most potent HETE to induce mPTP opening; however, it should be noted that 5-HETE did not significantly accumulate in either WT or HEPiPLA_2_γKO mouse liver during HF feeding (Fig. 3). Notably, 15-HETE was not able to promote significant mitochondrial swelling at early time points. Next, because we and others have previously reported that the level of 12-HETE in serum and/or tissues in certain disease states was increased from submicromolar to low micromolar concentrations (9, 15, 23), we performed a dose-response profile of the sensitivity of Ca^2+^-induced mitochondrial swelling at low micromolar (1–5 μM) concentrations of the most abundant eicosanoid in murine liver, 12-HETE. Calcium-dependent mitochondrial swelling was enhanced by concentrations of 12-HETE as low as 2.5 μM (Fig. 4B). Next, since nucleotides including ADP have been demonstrated to potently inhibit mPTP opening, we examined whether 12-HETE was effective at reversing the nucleotide-mediated inhibition of Ca^2+^-induced mitochondrial swelling. As anticipated, ADP dramatically inhibited Ca^2+^-induced mitochondrial swelling (Fig. 4C). Low micromolar concentration (1.25 μM) of 12-HETE markedly reversed ADP inhibition of mPTP opening in concentration-dependent manner (Fig. 4C). Importantly, the Ca^2+^-induced mitochondrial swelling mediated by 12-HETE was completely reversed by cyclosporine A indicative of a conventional cyclophilin D–mediated mPTP opening process (Fig. 4C).

### High-fat feeding markedly sensitizes WT, but not HEPiPLA_2_*γ*KO, hepatic mitochondrial PTP opening to Ca^2+^

**Fig. 5:**
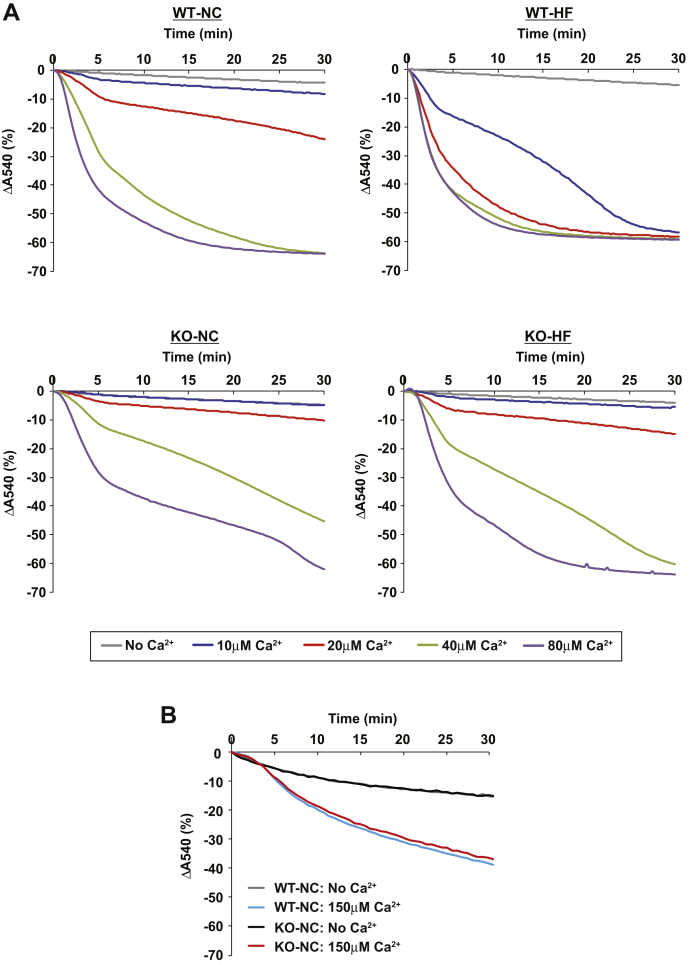
Comparison of the sensitivity of WT and HEPiPLA_2_γKO hepatic mitochondria to Ca^2+^-induced swelling following normal-chow versus high-fat diet feeding. A: Calcium sensitivity of mitochondrial swelling as examined in comparisons of WT versus HEPiPLA_2_γKO (KO) mice fed either a normal-chow diet (NC) or a high-fat diet (HF). Mitochondria isolated from WT and HEPiPLA_2_γKO mouse liver (NC vs. HF diet) were suspended in mitochondrial swelling buffer prior to initiation of swelling by Ca^2+^ (0, 10, 20, 40, or 80 μM final concentration). Mitochondrial swelling was monitored by measuring the decrease in absorbance at 540 nm. Representative tracings are shown from independent preparations of 5 mice each. B: Myocardial mitochondria were isolated from WT and HEPiPLA_2_γKO mouse hearts and mitochondrial swelling initiated with 150 μM Ca^2+^ was monitored by recording the decrease in absorbance at 540 nm. HEPiPLA_2_γKO, hepatocyte-specific iPLA_2_γ-knockout; iPLA_2_γ, calcium-independent phospholipase A_2_γ.

Next, we investigated whether HF feeding can alter the Ca^2+^-sensitivity of mPTP opening in hepatic mitochondria because of the upregulated eicosanoid content in liver. Accordingly, mitochondria were isolated from the livers of WT and HEPiPLA_2_γKO mice after NC or HF feeding, and mitochondrial PTP opening was monitored by measuring the degree of mitochondrial swelling at different concentrations of Ca^2+^ (i.e., 10, 20, 40, and 80 μM) (Fig. 5A). Interestingly, Ca^2+^-induced hepatic mitochondrial swelling was markedly attenuated in HEPiPLA_2_γKO mouse in comparison to WT controls regardless of whether the mice were fed a NC or an HF diet. However, as expected, myocardial mitochondria from HEPiPLA_2_γKO mice which contain normal amounts of iPLA_2_γ protein did not exhibit significant alterations in mPTP opening upon calcium challenge when compared with WT myocardial mitochondria (Fig. 5B). Importantly, wild-type hepatic mitochondrial swelling was greatly sensitized to low concentrations of Ca^2+^ after HF feeding such that these mitochondria were able to maximally swell in the presence of 10 μM Ca^2+^. In stark contrast, the Ca^2+^-sensitivity of the mPTP in hepatic mitochondria from HEPiPLA_2_γKO mice fed an HF diet was not significantly altered in the presence of 10 μM Ca^2+^ in comparison to those isolated from either WT or HEPiPLA_2_γKO mice fed a NC diet. At higher concentrations of Ca^2+^ (20–40 μM), mPTP opening in hepatic mitochondria from the HEPiPLA_2_γKO was only mildly increased by HF feeding when compared with NC-diet controls, thus representing a dramatic attenuation of calcium sensitivity in comparison to HF-fed WT liver mitochondria (Fig. 5A).

### High-fat feeding makes Ca^2+^-induced mPTP opening less resistant to ADP inhibition

**Fig. 6:**
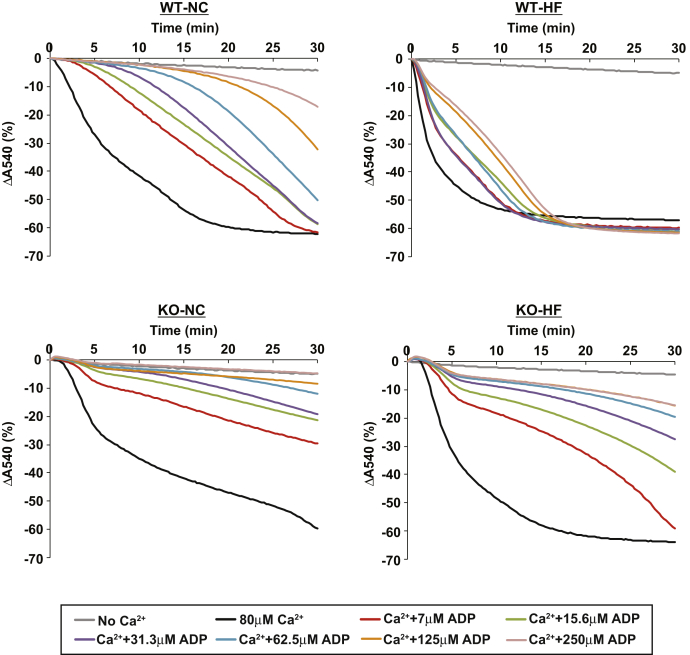
High-fat feeding dramatically decreases the potency of ADP inhibition of mPTP opening in WT, but not in HEPiPLA_2_γKO hepatic mitochondria. The ability of ADP to inhibit mitochondrial swelling in a concentration-dependent manner was examined with comparisons of isolated hepatic mitochondria from WT and HEPiPLA_2_γKO (KO) (∼6–7 month-old) mice fed either a normal-chow (NC) or high-fat (HF) diet. After resuspension of isolated mitochondria in mitochondrial swelling buffer containing the indicated concentrations of ADP, calcium (80 μM final concentration) was added to initiate mitochondrial swelling which was monitored by the decrease in absorbance at 540 nm. Representative tracings are shown from independent preparations of five mice for each condition. HEPiPLA_2_γKO, hepatocyte-specific iPLA_2_γ-knockout.

Nucleotides such as ATP and ADP are well-established potent inhibitors of mPTP opening through their binding to the adenine nucleotide translocase (ANT) (24). Because we found that liver mitochondria from HF-fed mice are more sensitive to mPTP opening at low concentrations of Ca^2+^, we hypothesized that HF feeding would desensitize the opening of mPTP to nucleotide inhibition. Based upon this hypothesis, we next examined the sensitivity of mPTP opening to ADP inhibition by measuring mitochondrial swelling at different ADP concentrations (Fig. 6). Interestingly, hepatic mitochondria from HF-fed WT mice were nearly completely resistant to ADP-mediated inhibition of mitochondrial swelling even at high concentrations (up to 250 μM) of ADP. By comparison, Ca^2+^-induced swelling of liver mitochondria from NC-fed WT mice was readily inhibited at low concentrations (7 μM) of ADP. However, hepatic mitochondria from HF-fed HEPiPLA_2_γKO mice remained effectively resistant to swelling in the presence of ADP. These results suggested that iPLA_2_γ-mediated generation of HETEs, which are greatly increased during HF feeding, desensitizes mPTP opening to ADP inhibition.

### Hepatic mitochondrial respiration is reduced in the presence of 12-HETE resulting in a decreased respiratory control ratio

**Fig. 7:**
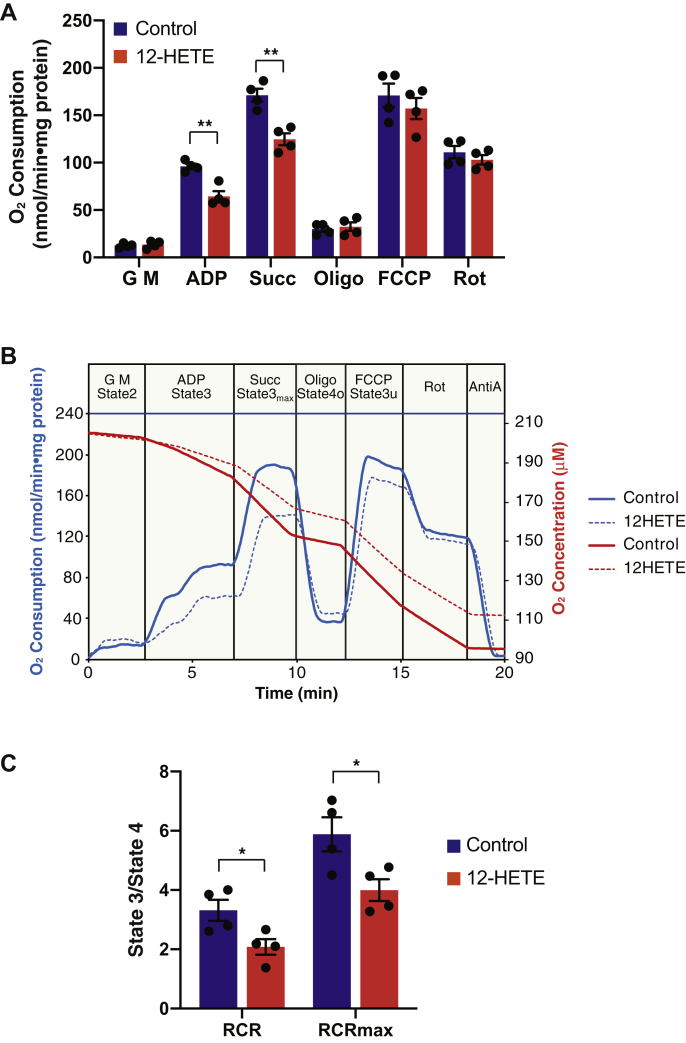
Inhibition of hepatic mitochondrial respiration by 12-HETE. High-resolution mitochondrial respirometry was performed using hepatic mitochondria isolated from wild-type mice fed a normal-chow diet to examine the effects of 12-HETE on respiratory states. A: Rate of oxygen consumption by mitochondria respired with glutamate/malate substrates (G M) (state 2) with sequential additions of ADP (state 3), succinate (Succ) (state 3_max_), oligomycin (Oligo) (state 4o), an oxidative phosphorylation uncoupler FCCP (state 3u), rotenone (Rot), and antimycin A (AntiA) in the absence (DMSO vehicle alone), or the presence of 5 μM 12-HETE. Net oxygen consumption rates were calculated by subtracting the respiration rate in the presence of antimycin A. The values are the means ± SEM from four independent high-resolution respirometry measurements. ∗∗*P* < 0.01 when compared with control. B: Representative tracings for oxygen consumption rate (blue lines) and oxygen concentration (red lines) in the absence (DMSO vehicle alone) or presence of 12-HETE during different respiration states are shown. C: Respiratory Control Ratio (RCR: state 3/state 4o) was calculated for comparisons in either the absence (control) versus presence of 12-HETE. RCR_max_ was calculated by the ratio of state 3_max_ to state 4o. ∗*P* < 0.05 in comparisons of control (DMSO vehicle alone) versus 12-HETE treatment. HETE, hydroxyeicosatetraenoic acid.

Next, we investigated the effect of 12-HETE on hepatic mitochondrial respiration. High resolution mitochondrial respirometry was performed utilizing liver mitochondria isolated from WT mice fed a NC diet. Oxygen consumption at each respiratory state was monitored by sequentially adding glutamate/malate (state 2), ADP (state 3), succinate (state 3_max_), oligomycin (state 4o), FCCP for uncoupling (state 3u), rotenone for complex I inhibition, and antimycin A in the absence or the presence of 12-HETE (Fig. 7). Hepatic mitochondrial respiration was significantly reduced in the presence of 12-HETE at ADP-driven state 3 and state 3_max_. In contrast, oligomycin-induced state 4 respiration (state 4o) was not significantly changed by addition of 12-HETE. These results demonstrate that the presence of 12-HETE partially inhibits ADP-stimulated mitochondrial oxygen consumption resulting in lowered respiratory control ratios (state 3/state 4o and state 3_max_/state 4o) when compared with nontreated control mitochondria, thereby implicating 12-HETE in mediating the observed disruption in mitochondrial respiratory function.

### Cellular calcium overload by calcium ionophore induces cytotoxicity in WT hepatocytes which is attenuated in HEPiPLA_2_*γ*KO hepatocytes

**Fig. 8:**
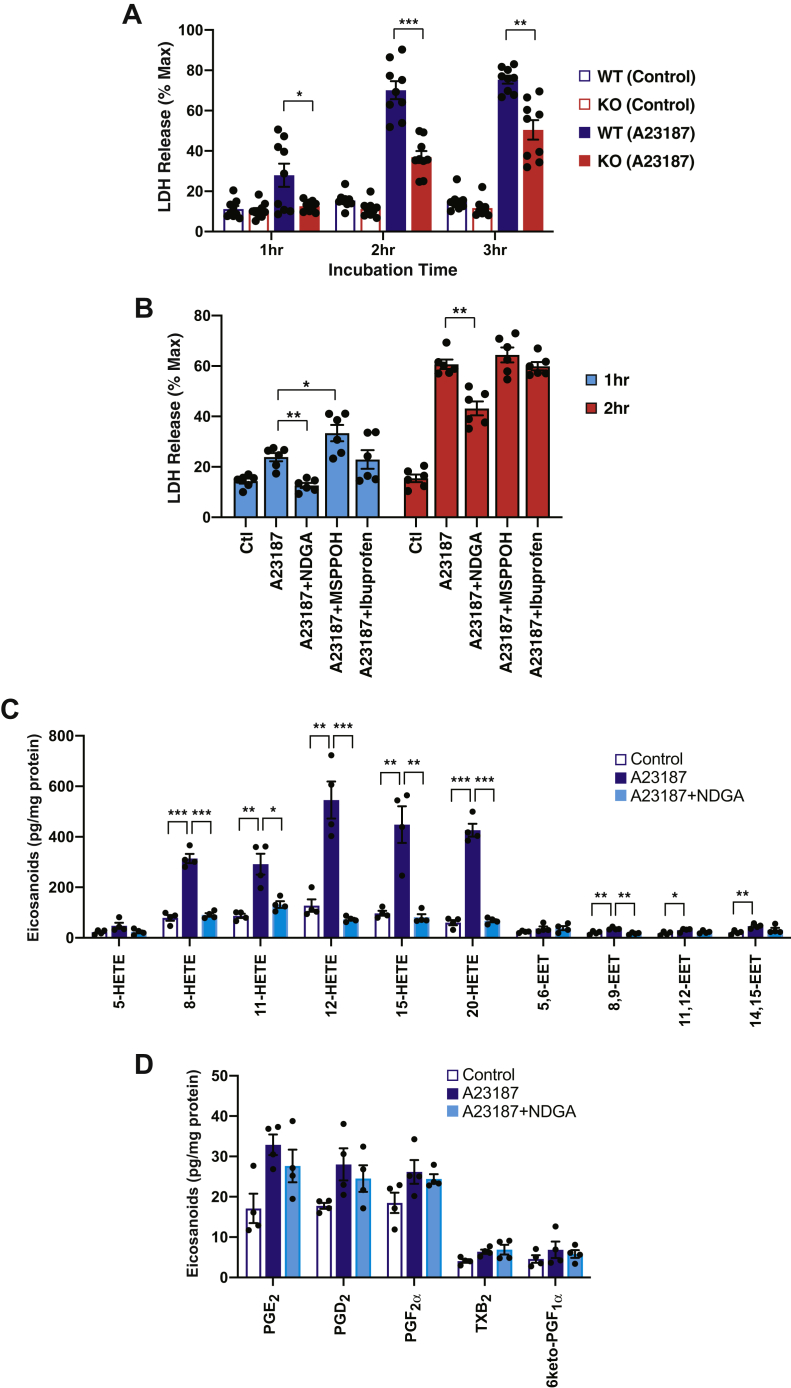
Release of lactate dehydrogenase (LDH) from primary WT and HEPiPLA_2_γKO hepatocytes following treatment with calcium ionophore. A: Primary hepatocytes from WT and HEPiPLA_2_γKO mice were isolated as described in Materials and Methods. Hepatocytes cultured in 12-well plates were exposed to either DMSO vehicle alone (0.1%, v/v) or 5 μM A23187 in fresh serum-free medium for the hours indicated followed by collection of the medium. LDH released into the cell medium (indicative of hepatocyte cell death) was quantified by performing an enzymatic assay of LDH activity present in the cell medium. ∗*P* < 0.05, ∗∗*P* < 0.001, and ∗∗∗*P* < 0.0001 when compared with WT stimulated with A23187 for the same incubation time. Values are the means of nine independent preparations with SEM. B: Primary hepatocytes from WT mice were stimulated with either DMSO vehicle alone (0.1% v/v) or 5 μM A23187 in serum-free media for the hours indicated in the presence of the following oxygenase inhibitors: 20 μM NDGA, 40 μM MSPPOH, or 10 μM ibuprofen for selective inhibition of lipoxygenases, cytochrome P450 epoxygenases, or cyclooxygenases, respectively. ∗*P* < 0.05 and ∗∗*P* < 0.001 when compared with the A23187 positive control. Values are expressed with means of the six independent preparations with SEM. Maximum cellular LDH activity released into extracellular space was determined after cell membrane disruption with 0.5% Triton X-100. C and D: Primary hepatocytes isolated from WT mice on a normal-chow diet were stimulated with either DMSO vehicle alone (control) or 5 μM A23187 in serum-free media for 30 min in the absence or presence of 20 μM NDGA. Cellular eicosanoids including HETEs, EETs (C), and prostaglandins (D) were determined and quantitated by LC-MS/MS as described under Materials and Methods. Values are expressed as the means of four independent preparations with SEM. ∗*P* < 0.05, ∗∗*P* < 0.01 and ∗∗∗*P* < 0.001. EET, epoxyeicosatrienoic acid; HEPiPLA_2_γKO, hepatocyte-specific iPLA_2_γ-knockout; HETE, hydroxyeicosatetraenoic acid; MSPPOH, N-(methylsulfonyl)-2-(2-propynyloxy)-benzenehexanamide; NDGA, nordihydroguaiaretic acid.

Diet-induced obesity causes pathologic cellular alterations such as disrupted cellular signaling, abnormal lipid metabolism, and mitochondrial dysfunction leading to cell death. Considering the marked iPLA_2_γ-dependent elevation of 12-HETE in murine liver following HF feeding and the ability of 12-HETE to inhibit mitochondrial respiration and enhance mPTP opening, we sought to test whether HEPiPLA_2_γKO hepatocytes were resistant to cell death relative to WT control hepatocytes. For this purpose, cytotoxicity was determined by quantifying the activity of LDH which is released into the media after induction of ER stress by stimulation with calcium ionophore A23187. Control WT primary hepatocytes obtained from NC-fed mice readily released LDH following a relatively short (1 h) incubation with A23187 (Fig. 8A). In contrast, HEPiPLA_2_γKO hepatocytes isolated from NC fed mice did not significantly release LDH into the media after 1 h incubation with A23187 when compared with nontreated controls. Longer exposure (2–3 h) of HEPiPLA_2_γKO hepatocytes to A23187 resulted in significantly lowered release of LDH activity in comparison to WT cells. Importantly, LDH release into extracellular space by calcium overload from WT hepatocytes was inhibited by the LOX inhibitor NDGA but not by the cytochrome P450 epoxygenase inhibitor N-(methylsulfonyl)-2-(2-propynyloxy)-benzenehexanamide nor by the COX-selective inhibitor ibuprofen (Fig. 8B). Supporting the results from pharmacologic inhibition assays, mass spectrometric analyses of intracellular eicosanoids showed that calcium ionophore activated the predominant production of various HETEs with modest increases in EETs, but not in prostaglandins, in primary hepatocytes (Fig. 8C, D). Treatment with NDGA nearly completely abolished A23187-activated production of LOX-mediated metabolites of AA (i.e., 8-, 11-, 12- and 15-HETEs) (Fig. 8C). Collectively, these results suggest that an iPLA_2_γ-LOX lipid metabolic axis which upregulates hepatic HETE production upon cellular stress during high fat diet–induced obesity is likely involved in hepatocyte cell death.

## Discussion

Previous work from our laboratory reported that global genetic ablation of iPLA_2_γ resulted in multicomponent abnormal phenotypes which included compromised mitochondrial ultrastructure and function, insulin hypersensitivity, the inability to gain weight during HF feeding, impaired skeletal muscular respiration and strength, and lower triacylglycerol content in adipose tissue (20). Furthermore, liver mitochondria from the germline iPLA_2_γ^−/−^ mouse displayed remarkable resistance to Ca^2+^-activated mPTP opening resulting in the prevention of cytochrome *c* release (18). Interestingly, our additional studies using cardiomyocyte-specific transgenic iPLA_2_γ mouse heart revealed that cardiac myocyte mitochondrial iPLA_2_γ can be activated by divalent cations (i.e., Ca^2+^ and Mg^2+^) (25). Moreover, cardiomyocyte-specific knockout of iPLA_2_γ demonstrated decreased infarct size after ischemia-reperfusion injury through attenuation of eicosanoid production and mPTP opening (15). In this study, we engineered and utilized a hepatocyte-specific iPLA_2_γ knockout mouse to investigate the distinct roles of hepatic iPLA_2_γ on cellular oxidized lipid metabolism and hepatocyte cell death using an HF-induced obesity model. By employing HF-fed HEPiPLA_2_γKO mice, we demonstrated that genetic ablation of hepatic iPLA_2_γ improved glucose tolerance and decreased production of detrimental oxidized arachidonate metabolites resulting in a decrease in Ca^2+^-induced mPTP opening. Intriguingly, an HF diet increased 12-HETE production in wild-type mice that subsequently amplified mPTP opening by sensitizing it to lower concentrations of Ca^2+^ and blocking ADP-mediated protection against Ca^2+^-induced mitochondrial swelling. Remarkably, hepatocyte-specific deletion of iPLA_2_γ resulted in markedly lower amounts of 12-HETE which minimized the deleterious effects of HF feeding. Finally, we demonstrate that either the absence of iPLA_2_γ (in HEPiPLA_2_γ KO hepatocytes) or pharmacological inhibition of LOXs (by NDGA) attenuates Ca^2+^-mediated cytotoxicity in hepatocytes emphasizing the importance of these interconnected lipid metabolic pathways in mediating responses to cellular stress.

The ability of eicosanoids to function as lipid second messengers in diverse (patho)physiological conditions has been extensively studied for decades (26). However, to the best of our knowledge, the ability of eicosanoids to mediate mPTP opening have not been previously examined. Importantly, this study demonstrates that the LOX product 12-HETE is the most predominant oxidized AA metabolite in liver and that it was dramatically elevated (∼2.5-fold) by an HF diet. A number of previous studies have reported multiple detrimental effects of LOX-generated HETE signaling which include induction of oxidative stress during cardiac ischemia/reperfusion and activation of proinflammatory effects via p38MAPK for immune responses by stimulation of cytokine and inflammatory gene expression presumably through activation of G protein-coupled receptor 31 (10, 27, 28, 29, 30). Moreover, 12-LOX expression levels and activity were demonstrated to be upregulated in multiple cell types in various disease states (10, 31, 32, 33). Published studies utilizing 12-LOX knockout mouse models and pharmacologic inhibition of 12-LOX reported protective outcomes that resulted from inactivation of 12-LOX, which include improved glucose tolerance and insulin sensitivity following HF feeding, resistance to the development of diabetes by increasing islet resistance to inflammatory cytokines such as tumor necrosis factor α and IL-1β, and reduction of infarct size in ischemic damage (14, 33, 34, 35, 36, 37). In support of these previous observations, the use of cell type–specific conditional genetic ablation of iPLA_2_γ in conjunction with high-resolution respirometry of isolated mitochondria and analysis of hepatic eicosanoids suggest that HF feeding induces 12-HETE–mediated mitochondrial dysfunction which is initiated by the activation of hepatic iPLA_2_γ to generate nonesterified AA or arachidonoyl-lysolipids which serve as substrate(s) for 12-LOX.

Mitochondrial membrane–associated calcium-independent PLA_2_γ can be activated by divalent cations or loss of membrane potential producing free AA and 2-AA-lysolipids (25, 38, 39). Recently, we have found that a variety of oxygenases (COXs and LOX) oxidize 2-AA-lysolipids as well as free AA, the products of which (i.e., oxidized lysophospholipids) can be further hydrolyzed by intracellular lysophospholipases to nonesterified oxidized AA metabolites (38, 40). Thus, in this context, iPLA_2_γ activation is a crucial step for providing AA-containing lipid precursors for downstream oxygenase enzymes. Furthermore, our recent study has demonstrated that production of 12-HETE/12-HETE lysophospholipids mediated by the combined actions of iPLA_2_γ and 12-LOX were increased in calcium-ionophore–activated platelets (40). Importantly, thrombin-activated platelets released nonesterified 12-HETE exclusively among various oxidized AA metabolites, and 12-HETE at nanomolar concentrations was demonstrated to induce the release of proinflammatory cytokines (i.e., tumor necrosis factor α and interleukin-8) from THP-1 monocytic cells (41). In addition, hepatic mitochondrial PTP opening as measured using the germline iPLA_2_γKO mouse was dramatically attenuated upon Ca^2+^ challenge preventing translocation of proapoptotic cytochrome *c* into the cytosol (18). Similar observations reported in this study utilizing the HEPiPLA_2_γKO mouse imply that iPLA_2_γ-mediated 12-HETE production exerts deleterious effects specifically in isolated hepatocytes leading to the progression of cell death likely via mPTP opening.

Established as one of the determinants for cell death, the mPTP has been the focus of numerous investigations to identify its protein composition, activation mechanism, regulatory activators/inhibitors, and its physiologic and pathologic roles in cell survival and death (42, 43). While Ca^2+^ is a necessary component for opening of the mPTP, additional activators of this process include inorganic phosphate, fatty acids, oxidative stress, carboxyatractyloside, adenine nucleotide depletion, and high pH (44). Despite impressive advances in characterizing the mPTP, the precise mechanism of pore opening and the structure of the pore complex are still not completely understood. One of the most intriguing observations in this study is that mitochondria from WT HF-fed mice were nearly completely desensitized to ADP-mediated inhibition of mPTP opening, which, in marked contrast, was not observed in the HEPiPLA_2_γKO mouse. More importantly, our finding that the inhibition of mPTP opening by ADP was reversed by exogenous 12-HETE implicates hepatic iPLA_2_γ-mediated 12-HETE production as a likely facilitator of cell death through disabling cellular protective mechanism(s) against irreversible pathologic opening of the mPTP. ADP has been demonstrated to bind to the mitochondrial ANT on both the cytoplasmic and matrix sides of the inner mitochondrial membrane thereby desensitizing the mPTP channel to calcium ion (45). The ADP/ATP translocase has also been shown to be one of the components of the mPTP complex and can activate mPTP opening through its interaction with mitochondrial matrix protein cyclophilin D (46, 47). Recently, Karch *et al.* (48) demonstrated that genetic ablation of various isoforms of ANT (i.e., *Ant1*, *Ant2*, and *Ant4*) resulted in loss of mPTP opening activity. Moreover, other studies have reported that the cyclophilin D knockout mouse exhibited improvement of HF-induced glucose intolerance (49, 50). However, the exact mechanism for the ADP-mediated inhibition of mPTP channel opening via conformational changes through the interaction between ANT and ADP is still not clearly understood. Our results suggest that 12-HETE regulates the affinity of ADP for the inhibitory binding site of ANT in a similar manner as (carboxy)atractylate antagonizes ADP-mediated inhibition of mPTP opening through binding the *c*-conformation of ANT present in the cytoplasmic mitochondrial membrane (51). This proposed mechanism also suggests that inhibition of ADP/ATP translocase activity by 12-HETE is likely responsible for the attenuation of ADP-induced state 3 mitochondrial respiration that we observed in this study.

Collectively, the results of this study demonstrate that hepatic iPLA_2_γ plays central roles in HF diet–induced pathologic alterations by providing AA and/or AA-lysophospholipids to 12-LOX for detrimental 12-HETE production that promotes the opening probability of the mPTP and disrupts mitochondrial bioenergetics leading to metabolic stress and the initiation of cell death.

## Data availability

All data described in this study are contained in the manuscript.

## Conflict of interest

The authors declare that they have no conflicts of interest with the contents of this article.
